# Supplementary figures and images for: Computational pan-genome mapping and pairwise SNP-distance improve detection of Mycobacterium tuberculosis transmission clusters
Source: PLoS Comput Biol. 2019 Dec 9;15(12):e1007527. doi: 10.1371/journal.pcbi.1007527 (PMC6922483; doi:10.1371/journal.pcbi.1007527)

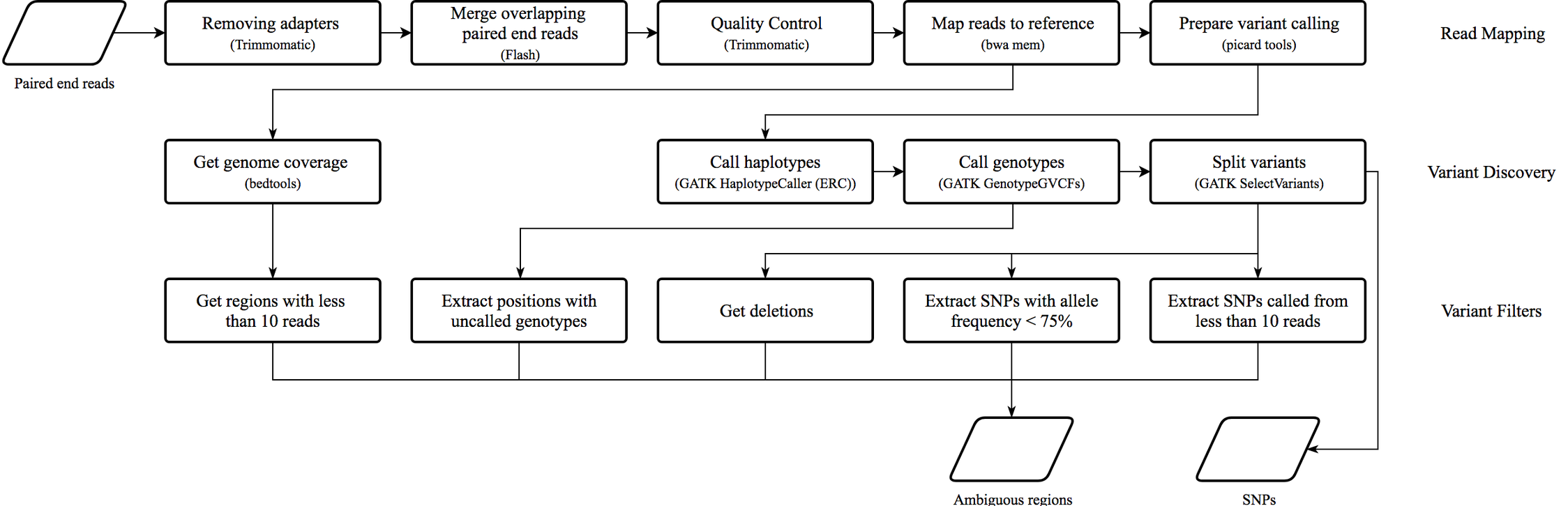

Supplement: S1 Fig — We divide the task in three parts: read mapping, variant calling and filtering of variants and detection of low-quality regions. We prepare the reads by removing sequence adapters and merging overlapping paired end reads. After that low-quality reads are filtered and reads with ends of low base quality are trimmed. High-quality reads are mapped to a reference and necessary steps for variant calling are taken. We use GATK [55] for variant calling and bedtools [58] to calculate the coverage of the genome. The results are filtered to detect regions of low-quality and high-quality SNPs. (TIF) [file pcbi.1007527.s002.tif]
