# Supplementary material for: Computational pan-genome mapping and pairwise SNP-distance improve detection of Mycobacterium tuberculosis transmission clusters
Source: PLoS Comput Biol. 2019 Dec 9;15(12):e1007527. doi: 10.1371/journal.pcbi.1007527 (PMC6922483; doi:10.1371/journal.pcbi.1007527)
Supplement: S1 Table — (PDF) [file pcbi.1007527.s003.pdf]

**S1 Table. Description of clusters in the simulation dataset.**

| Cluster | *M. tuberculosis* strain | number of samples | number of inter-cluster SNPs | number of intra-cluster SNPs |
|---|---|---|---|---|
| C1 | H37Rv | 19 | 36 | 29 |
| C2 | H37Rv | 7 | 29 | 11 |
| C3 | H37Rv | 8 | 32 | 12 |
| C4 | H37Rv | 31 | 13 | 47 |
| C5 | H37Rv | 9 | 6 | 14 |
| C6 | MDRMA2082 | 18 | 30 | 27 |
| C7 | MDRMA2082 | 30 | 31 | 45 |
| C8 | MDRMA2082 | 11 | 15 | 17 |
| C9 | MDRMA2082 | 11 | 20 | 17 |
| C10 | MDRMA2082 | 8 | 14 | 12 |
| C11 | HKBS1 | 4 | 40 | 11 |
| C12 | HKBS1 | 3 | 37 | 11 |
| C13 | HKBS1 | 16 | 27 | 24 |
| C14 | HKBS1 | 20 | 27 | 30 |
| C15 | HKBS1 | 22 | 40 | 33 |
| C16 | TB282 | 15 | 16 | 23 |
| C17 | TB282 | 4 | 35 | 11 |
| C18 | TB282 | 51 | 27 | 77 |
| C19 | TB282 | 11 | 31 | 17 |
| C20 | TB282 | 25 | 31 | 38 |
