# Supplementary material for: Computational pan-genome mapping and pairwise SNP-distance improve detection of Mycobacterium tuberculosis transmission clusters
Source: PLoS Comput Biol. 2019 Dec 9;15(12):e1007527. doi: 10.1371/journal.pcbi.1007527 (PMC6922483; doi:10.1371/journal.pcbi.1007527)
Supplement: S2 Table — Base differences were counted in a whole-genome alignment of the four genomes. Upper triangular part of table shows hamming distance of sequences in WGA ignoring gaps, while the lower part of the table lists all differences, including gaps. (PDF) [file pcbi.1007527.s004.pdf]

**S2 Table. Description of genomes used for the simulation dataset.**

| | H37Rv | MDRMA2082 | HKBS1 | TB282 |
|---|---|---|---|---|
| H37Rv | 0 | 1046 | 2321 | 2508 |
| MDRMA2082 | 1279 | 0 | 2273 | 2461 |
| HKBS1 | 104466 | 104367 | 0 | 408 |
| TB282 | 132850 | 132766 | 41429 | 0 |

Base differences were counted in a whole-genome alignment of the four genomes. Upper triangular part of table shows hamming distance of sequences in WGA ignoring gaps, while the lower part of the table lists all differences, including gaps.
