# Supplementary material for: Computational pan-genome mapping and pairwise SNP-distance improve detection of Mycobacterium tuberculosis transmission clusters
Source: PLoS Comput Biol. 2019 Dec 9;15(12):e1007527. doi: 10.1371/journal.pcbi.1007527 (PMC6922483; doi:10.1371/journal.pcbi.1007527)
Supplement: S3 Table — We count the simulated SNPs in all simulated samples located on genome specific regions that are not part of the H37Rv genome. (PDF) [file pcbi.1007527.s005.pdf]

S3 Table. Comparison of genomes used in the simulation dataset to the *M. tuberculosis* strain.

	inter-cluster SNPs	intra-cluster SNPs
MDRMA2082	0	1
HKBS1	4	3
TB282	8	9

We count the simulated SNPs in all simulated samples located on genome specific regions that are not part of the H37Rv genome.
