# Supplementary material for: Computational pan-genome mapping and pairwise SNP-distance improve detection of Mycobacterium tuberculosis transmission clusters
Source: PLoS Comput Biol. 2019 Dec 9;15(12):e1007527. doi: 10.1371/journal.pcbi.1007527 (PMC6922483; doi:10.1371/journal.pcbi.1007527)
Supplement: S4 Table — provides the counts for transmission cluster links for the exclusion, substitution and pairwise method for mappings to the M. tuberculosis H37Rv strain and the computational pan-genome. (PDF) [file pcbi.1007527.s006.pdf]

TRUTH	C1	C2	C3	C4	C5	C6	C7	C8	C9	C10	C11	C12	C13	C14	C15	C16	C17	C18	C19	C20
transmission links intra-cluster	127	21	28	287	36	125	205	54	52	28	6	3	101	158	172	95	6	837	53	210
no relation intra-cluster	44	0	0	178	0	28	230	1	3	0	0	0	19	32	59	10	0	438	2	90

**Method**	**Metric**	**C1**	**C2**	**C3**	**C4**	**C5**	**C6**	**C7**	**C8**	**C9**	**C10**	**C11**	**C12**	**C13**	**C14**	**C15**	**C16**	**C17**	**C18**	**C19**	**C20**
Exclusion	TP	127	21	28	287	36	125	205	54	52	28	6	3	101	158	172	95	6	837	53	210
	TN	0	0	0	0	0	0	0	0	0	0	0	0	0	0	0	0	0	0	0	0
	FP	44	0	0	178	0	28	230	1	3	0	0	0	19	32	59	10	0	438	2	90
	FN	0	0	0	0	0	0	0	0	0	0	0	0	0	0	0	0	0	0	0	0
Substitution	TP	106	21	28	272	36	2	0	0	0	0	0	0	0	0	0	0	0	0	0	0
	TN	44	0	0	173	0	28	230	1	3	0	0	0	19	32	59	10	0	438	2	90
	FP	0	0	0	5	0	0	0	0	0	0	0	0	0	0	0	0	0	0	0	0
	FN	21	0	0	15	0	123	205	54	52	28	6	3	101	158	172	95	6	837	53	210
PANPASCO	TP	125	21	28	279	36	125	170	54	48	28	6	3	99	149	168	95	6	829	53	203
	TN	35	0	0	172	0	15	225	0	3	0	0	0	6	31	49	6	0	300	2	65
	FP	9	0	0	6	0	13	5	1	0	0	0	0	13	1	10	4	0	138	0	25
	FN	2	0	0	8	0	0	35	0	4	0	0	0	2	9	4	0	0	8	0	7

TP = true positives, TN = true negatives, FP = false positives, FN = false negatives
