# Supplementary material for: Computational pan-genome mapping and pairwise SNP-distance improve detection of Mycobacterium tuberculosis transmission clusters
Source: PLoS Comput Biol. 2019 Dec 9;15(12):e1007527. doi: 10.1371/journal.pcbi.1007527 (PMC6922483; doi:10.1371/journal.pcbi.1007527)
Supplement: S5 Table — We used the all three methods (exclusion, substitution, PANPASCO) with the commonly used M. tuberculosis H37Rv and the computational pan-genome reference genomes for classification of links between samples in the simulated dataset. (PDF) [file pcbi.1007527.s007.pdf]

S5 Table. Comparison of SNP-counting methods in all clusters of the simulation dataset.

Method	Reference genome	Sensitivity	Specificity	Accuracy	F-Score
H37Rv	exclusion	**1.000**	0.782	0.793	0.326
	substitution	0.179	**1.000**	0.959	0.303
	PANPASCO	0.896	0.998	**0.993**	**0.930**
pan-genome	exclusion	**1.000**	0.782	0.793	0.326
	substitution	0.000	**1.000**	0.950	0.000
	PANPASCO	0.970	0.995	**0.994**	**0.943**
