# Supplementary material for: Computational pan-genome mapping and pairwise SNP-distance improve detection of Mycobacterium tuberculosis transmission clusters
Source: PLoS Comput Biol. 2019 Dec 9;15(12):e1007527. doi: 10.1371/journal.pcbi.1007527 (PMC6922483; doi:10.1371/journal.pcbi.1007527)
Supplement: S6 Table — contains the full SNP matrix for all samples in the UKTB7 dataset. (PDF) [file pcbi.1007527.s008.pdf]

Published order	1	2	3	4	5	6	7	8	9	10	11	12	13	14	15	16	17			
Year of sample isolation	2008	2007	2008	2006	2006	2007	2002	2004	2004	2005	2011	2004	2011	2011	2008	2007	2005			
ENA id	ERR046769	ERR046942	ERR046770	ERR038298	ERR040087	ERR046940	ERR039323	ERR046729	ERR046772	ERR046914	ERR192249	ERR040108	ERR192250	ERR040090	ERR046771	ERR046773	ERR040088			
Published name	P027b	P026c	P026d	P027a	P026a	P026b	P076a	P076b	P076c	P076d	P334	P174	P335	P175	P066	P211	P037	Locus on pan-genome	Locus on H37Rv strain	In Walker et al. 2013
	X	X	G	X	X	X	X	X	G	X	C	X	C	X	G	X	X	42168	39017	
	G	G	G	T	T	T	T	T	T	T	T	T	T	T	T	T	T	170358	166624	yes
	C	C	C	C	C	C	C	C	C	C	C	C	G	C	C	C	C	514289	468370	yes
	C	C	C	C	C	C	C	C	C	C	C	C	C	C	C	C	T	682920	632594	yes
	X	C	C	X	C	X	C	X	X	C	C	G	C	C	X	C	C	939618	873144	
	X	C	C	X	C	X	C	X	X	C	C	G	C	C	X	C	C	939619	873145	
	G	G	G	G	G	G	X	G	X	X	G	G	A	A	A	A	G	946431	879955	yes
	C	C	C	C	C	C	C	C	C	C	C	C	C	C	C	C	T	969914	899416	yes
	T	T	T	T	T	T	C	C	C	C	C	C	C	C	C	C	C	1023003	945783	yes
	A	A	A	A	A	A	G	G	G	G	G	G	G	G	G	G	G	1167980	1085807	yes
	G	G	G	G	G	G	G	G	G	G	G	G	X	C	G	G	G	1179475	1096470	
	C	C	C	C	C	C	C	C	C	C	C	C	C	G	C	C	C	1179513	1096508	
	T	T	T	T	T	T	T	T	T	T	T	T	T	C	T	T	T	1179515	1096510	
	X	X	X	X	G	X	X	X	T	X	X	X	X	X	X	X	X	1260326	-	
	X	G	G	X	C	G	X	G	G	G	G	X	G	X	X	X	X	1375328	1284071	
	G	G	G	G	G	G	X	X	X	G	C	C	G	G	G	G	G	1466454	1375047	yes
	G	G	G	G	G	G	X	X	X	G	C	C	G	G	G	G	G	1466455	1375048	yes
	X	X	C	X	C	X	G	C	X	X	X	X	X	X	X	C	X	1651259	-	
	C	C	C	C	C	C	C	C	C	C	C	C	C	C	C	C	T	1691909	1584192	yes
	A	A	A	A	A	A	G	G	G	G	G	G	G	G	G	G	G	2160699	1897180	yes
	A	A	A	A	A	A	A	A	A	A	A	A	A	A	A	A	G	2404646	2096225	yes
	C	C	C	C	C	C	C	C	C	C	T	C	C	C	C	C	C	2649456	2303882	yes
	G	G	G	G	G	G	A	A	A	A	A	A	A	A	A	A	G	2738716	2383755	yes
	X	G	X	C	X	G	C	X	X	X	G	X	G	X	X	X	X	2768703	2413615	
	C	C	C	C	C	C	C	C	C	C	C	C	G	C	C	C	C	2897425	2537713	yes
	G	G	G	G	G	G	G	G	G	G	G	G	G	G	G	G	A	3261991	2854386	yes
	X	X	X	X	X	X	X	X	X	X	T	X	X	X	X	X	C	3362566	2945072	
	C	C	C	X	G	C	X	C	C	X	C	X	X	C	C	C	X	3896906	3426077	
	C	X	C	X	X	X	X	X	X	X	G	X	X	C	X	X	X	4386001	-	
	C	C	C	C	C	C	C	C	C	C	T	C	C	C	C	C	C	4814383	4120151	yes
	G	G	G	G	G	G	G	G	G	G	G	G	G	G	G	G	A	4852391	4156479	yes
	G	G	G	G	G	G	G	G	G	G	G	G	G	G	G	C	G	4946995	4250896	yes
	G	G	G	G	G	G	G	G	G	G	G	G	G	G	T	G	G	4993297	4297167	
	X	C	X	C	C	X	C	X	X	X	C	C	X	C	X	G	C	5082697	-	
	G	G	G	G	G	G	X	G	X	X	G	G	C	C	C	C	G	5096147	4398748	yes
	X	X	X	X	G	X	X	X	X	X	C	X	X	X	X	X	X	5141599	-	
