# Supplementary material for: Computational pan-genome mapping and pairwise SNP-distance improve detection of Mycobacterium tuberculosis transmission clusters
Source: PLoS Comput Biol. 2019 Dec 9;15(12):e1007527. doi: 10.1371/journal.pcbi.1007527 (PMC6922483; doi:10.1371/journal.pcbi.1007527)
Supplement: S7 Table — provides detailed description of the genomes used for building the computational M. tuberculosis pan-genome including accession numbers, sort order and lineage. (PDF) [file pcbi.1007527.s009.pdf]

Assembly Accession	ASM Name	Accession number	Description	Position in pan-genome	Length of genome	Lineage	Used for simulation
GCF_001708265.1	ASM170826v1	NZ_CP016888.1	Mycobacterium tuberculosis strain SCAID 252.0 chromosome, complete genome	1	4439387	Beijing	
GCF_000331445.1	ASM33144v1	NC_020089.1	Mycobacterium tuberculosis 7199-99 complete genome	2	4421197	Haarlem	
GCF_002116775.1	ASM211677v1	NZ_CP017594.1	Mycobacterium tuberculosis strain Beijing-like/36918 chromosome, complete genome	3	4441591	Beijing	
GCF_000153685.2	ASM15368v2	NC_022350.1	Mycobacterium tuberculosis str. Haarlem, complete genome	4	4408224	Haarlem	
GCF_002072775.2	ASM207277v2	NZ_CP020381.2	Mycobacterium tuberculosis strain MTB1, complete genome	5	4433542	New-1	
GCF_002116755.1	ASM211675v1	NZ_CP017593.1	Mycobacterium tuberculosis strain Beijing-like/35049 chromosome, complete genome	6	4427062	Beijing	
GCF_000016925.1	ASM1692v1	NC_009565.1	Mycobacterium tuberculosis F11, complete genome	7	4424435	LAM	
GCF_002116835.1	ASM211683v1	NZ_CP017597.1	Mycobacterium tuberculosis strain Beijing-like/50148 chromosome, complete genome	8	4444417	Beijing	
GCF_002208235.1	ASM220823v1	NZ_CP022014.1	Mycobacterium tuberculosis strain MTB2 chromosome, complete genome	9	4417716	Beijing	
GCF_002116795.1	ASM211679v1	NZ_CP017595.1	Mycobacterium tuberculosis strain Beijing-like/38774 chromosome, complete genome	10	4431885	Beijing	
GCF_002357955.1	ASM235795v1	NZ_AP018035.1	Mycobacterium tuberculosis DNA, complete genome, strain: HN-321	11	4421540	Beijing	
GCF_000786505.1	MT49-02	NZ_HG813240.1	Mycobacterium tuberculosis 49-02 complete genome	12	4412379	Beijing	
GCF_001938725.1	ASM193872v1	NZ_CP016972.1	Mycobacterium tuberculosis H37Ra chromosome, complete genome	13	4426109	Euro-American (4.9)	
GCF_001545015.1	ASM154501v1	NZ_CP010339.1	Mycobacterium tuberculosis strain 22103, complete genome	14	4399422	Euro-American (4.2)	
GCF_002357935.1	ASM235793v1	NZ_AP018034.1	Mycobacterium tuberculosis DNA, complete genome, strain: HN-205	15	4411033	Beijing	
GCF_001544705.1	ASM154470v1	NZ_CP010330.1	Mycobacterium tuberculosis strain F28, complete genome	16	4421903	Euro-American (4.9)	
GCF_000400615.1	ASM40061v1	NC_021251.1	Mycobacterium tuberculosis CCDC5079, complete genome	17	4414325	Beijing	
GCF_002356255.1	ASM235625v1	NZ_AP018033.1	Mycobacterium tuberculosis DNA, complete genome, strain: HN-024	18	4399916	EAI	
GCF_002886865.1	ASM288686v1	NZ_CP025607.1	Mycobacterium tuberculosis strain GG-186-10 chromosome, complete genome	19	4411478	Haarlem	
GCF_000572195.1	ASM57219v1	NZ_CP002885.1	Mycobacterium tuberculosis CCDC5180, complete genome	20	4414346	Beijing	

GCF_000572155.1	ASM57215v1	NZ_CP002882.1	Mycobacterium tuberculosis BT2, complete genome	21	4401899 Beijing	
GCF_002887145.1	ASM288714v1	NZ_CP025599.1	Mycobacterium tuberculosis strain GG-45-11 chromosome, complete genome	22	4411469 Euro-American (4.8)	
GCF_002886945.1	ASM288694v1	NZ_CP025596.1	Mycobacterium tuberculosis strain GG-27-11 chromosome, complete genome	23	4411443 X-type	
GCF_002886165.1	ASM288616v1	NZ_CP025594.1	Mycobacterium tuberculosis strain GG-5-10 chromosome, complete genome	24	4411442 Euro-American (4.8)	
GCF_000277735.2	ASM27773v2	NC_018143.2	Mycobacterium tuberculosis H37Rv, complete genome	25	4411709 Euro-American (4.9)	
GCF_002886405.1	ASM288640v1	NZ_CP025604.1	Mycobacterium tuberculosis strain GG-129-11 chromosome, complete genome	26	4411413 Euro-American (4.1.1.3)	
GCF_002886585.1	ASM288658v1	NZ_CP025608.1	Mycobacterium tuberculosis strain GG-229-10 chromosome, complete genome	27	4411519 LAM	
GCF_001922485.1	ASM192248v1	NZ_CP018778.1	Mycobacterium tuberculosis strain DK9897, complete genome	28	4411511 LAM	
GCF_002886195.1	ASM288619v1	NZ_CP025595.1	Mycobacterium tuberculosis strain GG-20-11 chromosome, complete genome	29	4411504 LAM	
GCF_002886145.1	ASM288614v1	NZ_CP025593.1	Mycobacterium tuberculosis strain GG-111-10 chromosome, complete genome	30	4411563 Haarlem	
GCF_002886335.1	ASM288633v1	NZ_CP025601.1	Mycobacterium tuberculosis strain GG-90-10 chromosome, complete genome	31	4411602 LAM	
GCF_000016145.1	ASM1614v1	NC_009525.1	Mycobacterium tuberculosis H37Ra, complete genome	32	4419977 Euro-American (4.9)	
GCF_002357975.1	ASM235797v1	NZ_AP018036.1	Mycobacterium tuberculosis DNA, complete genome, strain: HN-506	33	4413362 Beijing	
GCF_000738475.1	ASM73847v1	NZ_CP009101.1	Mycobacterium tuberculosis strain ZMC13-88, complete genome	34	4411515 Beijing	
GCF_002887065.1	ASM288706v1	NZ_CP025597.1	Mycobacterium tuberculosis strain 36-11 chromosome, complete genome	35	4411469 Euro-American (4.8)	
GCF_000195955.2	ASM19595v2	NC_000962.3	Mycobacterium tuberculosis H37Rv, complete genome	36	4411532 Euro-American (4.9)	yes - H37Rv
GCF_002886225.1	ASM288622v1	NZ_CP025600.1	Mycobacterium tuberculosis strain 77-11 chromosome, complete genome	37	4411508 LAM	
GCF_002887255.1	ASM288725v1	NZ_CP025602.1	Mycobacterium tuberculosis strain GG-109-10 chromosome, complete genome	38	4411463 LAM	
GCF_000738445.1	ASM73844v1	NZ_CP009100.1	Mycobacterium tuberculosis strain ZMC13-264, complete genome	39	4411507 Beijing	
GCF_000827085.1	ASM82708v1	NZ_CP007027.1	Mycobacterium tuberculosis H37RvSiena, complete genome	40	4410911 Euro-American (4.9)	
GCF_002886685.1	ASM288668v1	NZ_CP025598.1	Mycobacterium tuberculosis strain GG-37-11 chromosome, complete genome	41	4411526 LAM	

GCF_002886775.1	ASM288677v1	NZ_CP025603.1	Mycobacterium tuberculosis strain GG-121-10 chromosome, complete genome	42	4411510	LAM	
GCF_002887335.1	ASM288733v1	NZ_CP025606.1	Mycobacterium tuberculosis strain GG-137-10 chromosome, complete genome	43	4411446	LAM	
GCF_000572125.1	ASM57212v1	NZ_CP002871.1	Mycobacterium tuberculosis HKBS1, complete genome	44	4407929	Beijing	yes - HKBS1
GCF_002447575.1	ASM244757v1	NZ_CP023608.1	Mycobacterium tuberculosis strain LE410 chromosome, complete genome	45	4411365	Euro-American (4.8)	
GCF_002447735.1	ASM244773v1	NZ_CP023616.1	Mycobacterium tuberculosis strain LN3668 chromosome, complete genome	46	4411494	Euro-American (4.8)	
GCF_002886505.1	ASM288650v1	NZ_CP025605.1	Mycobacterium tuberculosis strain GG-134-11 chromosome, complete genome	47	4411399	LAM	
GCF_002448095.1	ASM244809v1	NZ_CP023634.1	Mycobacterium tuberculosis strain TBDM2487 chromosome, complete genome	48	4411314	Euro-American (4.8)	
GCF_002447475.1	ASM244747v1	NZ_CP023603.1	Mycobacterium tuberculosis strain LE63 chromosome, complete genome	49	4411415	Euro-American (4.9)	
GCF_002447695.1	ASM244769v1	NZ_CP023614.1	Mycobacterium tuberculosis strain LN3588 chromosome, complete genome	50	4411379	LAM	
GCF_002447515.1	ASM244751v1	NZ_CP023605.1	Mycobacterium tuberculosis strain LE79 chromosome, complete genome	51	4411475	Haarlem	
GCF_002447975.1	ASM244797v1	NZ_CP023628.1	Mycobacterium tuberculosis strain MDRMA2082 chromosome, complete genome	52	4411479	Haarlem	yes - MDRRMA2082
GCF_002448155.1	ASM244815v1	NZ_CP023637.1	Mycobacterium tuberculosis strain TBDM2717 chromosome, complete genome	53	4411442	Haarlem	
GCF_002447755.1	ASM244775v1	NZ_CP023617.1	Mycobacterium tuberculosis strain LN3672 chromosome, complete genome	54	4411335	LAM	
GCF_002447995.1	ASM244799v1	NZ_CP023629.1	Mycobacterium tuberculosis strain MDRMA2260 chromosome, complete genome	55	4411352	LAM	
GCF_002446995.1	ASM244699v1	NZ_CP023579.1	Mycobacterium tuberculosis strain LE492 chromosome, complete genome	56	4411208	LAM	
GCF_002448135.1	ASM244813v1	NZ_CP023636.1	Mycobacterium tuberculosis strain TBDM2699 chromosome, complete genome	57	4411457	Haarlem	
GCF_002447715.1	ASM244771v1	NZ_CP023615.1	Mycobacterium tuberculosis strain LN3589 chromosome, complete genome	58	4411392	Haarlem	
GCF_002448015.1	ASM244801v1	NZ_CP023630.1	Mycobacterium tuberculosis strain MDRMA2441 chromosome, complete genome	59	4411454	Euro-American (4.7)	
GCF_002447195.1	ASM244719v1	NZ_CP023589.1	Mycobacterium tuberculosis strain TBV5000 chromosome, complete genome	60	4411318	LAM	
GCF_002448175.1	ASM244817v1	NZ_CP023638.1	Mycobacterium tuberculosis strain TBV4766 chromosome, complete genome	61	4411310	LAM	
GCF_002447775.1	ASM244777v1	NZ_CP023618.1	Mycobacterium tuberculosis strain LN3695 chromosome, complete genome	62	4411449	Haarlem	

GCF_002447415.1	ASM244741v1	NZ_CP023600.1	Mycobacterium tuberculosis strain CSV3611 chromosome, complete genome	63	4411439	Euro-American (4.7)
GCF_002447655.1	ASM244765v1	NZ_CP023612.1	Mycobacterium tuberculosis strain LN2978 chromosome, complete genome	64	4411331	Euro-American (4.9)
GCF_002448115.1	ASM244811v1	NZ_CP023635.1	Mycobacterium tuberculosis strain TBDM2489 chromosome, complete genome	65	4411369	LAM
GCF_002446875.1	ASM244687v1	NZ_CP023573.1	Mycobacterium tuberculosis strain CSV4519 chromosome, complete genome	66	4411288	LAM
GCF_002447215.1	ASM244721v1	NZ_CP023590.1	Mycobacterium tuberculosis strain TBV5362 chromosome, complete genome	67	4411331	LAM
GCF_002447895.1	ASM244789v1	NZ_CP023624.1	Mycobacterium tuberculosis strain MDRMA701 chromosome, complete genome	68	4411338	LAM
GCF_002447955.1	ASM244795v1	NZ_CP023627.1	Mycobacterium tuberculosis strain MDRMA2019 chromosome, complete genome	69	4411229	LAM
GCF_002448075.1	ASM244807v1	NZ_CP023633.1	Mycobacterium tuberculosis strain TBDM2444 chromosome, complete genome	70	4411284	LAM
GCF_002447455.1	ASM244745v1	NZ_CP023602.1	Mycobacterium tuberculosis strain LE13 chromosome, complete genome	71	4411412	Haarlem
GCF_002448035.1	ASM244803v1	NZ_CP023631.1	Mycobacterium tuberculosis strain TBDM1506 chromosome, complete genome	72	4411157	Euro-American (4.1.1)
GCF_002448055.1	ASM244805v1	NZ_CP023632.1	Mycobacterium tuberculosis strain TBDM2189 chromosome, complete genome	73	4411316	Euro-American (4.9)
GCF_000706665.1	ASM70666v1	NZ_CP007809.1	Mycobacterium tuberculosis strain KIT87190, complete genome	74	4410788	Beijing
GCF_002447015.1	ASM244701v1	NZ_CP023580.1	Mycobacterium tuberculosis strain LN180 chromosome, complete genome	75	4411436	Haarlem
GCF_002447855.1	ASM244785v1	NZ_CP023622.1	Mycobacterium tuberculosis strain MDRDM827 chromosome, complete genome	76	4411315	LAM
GCF_002446895.1	ASM244689v1	NZ_CP023574.1	Mycobacterium tuberculosis strain CSV4644 chromosome, complete genome	77	4411271	LAM
GCF_002446935.1	ASM244693v1	NZ_CP023576.1	Mycobacterium tuberculosis strain CSV10399 chromosome, complete genome	78	4411180	Euro-American (4.1.1.3)
GCF_002446975.1	ASM244697v1	NZ_CP023578.1	Mycobacterium tuberculosis strain LE486 chromosome, complete genome	79	4411180	LAM
GCF_002447155.1	ASM244715v1	NZ_CP023587.1	Mycobacterium tuberculosis strain ME1473 chromosome, complete genome	80	4411217	LAM
GCF_002447255.1	ASM244725v1	NZ_CP023592.1	Mycobacterium tuberculosis strain SLM036 chromosome, complete genome	81	4411342	LAM
GCF_002447295.1	ASM244729v1	NZ_CP023594.1	Mycobacterium tuberculosis strain SLM056 chromosome, complete genome	82	4411306	LAM
GCF_002447635.1	ASM244763v1	NZ_CP023611.1	Mycobacterium tuberculosis strain LN763 chromosome, complete genome	83	4411321	LAM

GCF_002447795.1	ASM244779v1	NZ_CP023619.1	Mycobacterium tuberculosis strain LN1100 chromosome, complete genome	84	4411392	Haarlem
GCF_002448215.1	ASM244821v1	NZ_CP023640.1	Mycobacterium tuberculosis strain TBV4952 chromosome, complete genome	85	4411414	Haarlem
GCF_002356015.1	ASM235601v1	NZ_AP017901.1	Mycobacterium tuberculosis DNA, complete genome, strain: NCGM946K2	86	4380602	LAM
GCF_002447035.1	ASM244703v1	NZ_CP023581.1	Mycobacterium tuberculosis strain LN2358 chromosome, complete genome	87	4411353	LAM
GCF_002447115.1	ASM244711v1	NZ_CP023585.1	Mycobacterium tuberculosis strain MDRDM1098 chromosome, complete genome	88	4411148	LAM
GCF_002447175.1	ASM244717v1	NZ_CP023588.1	Mycobacterium tuberculosis strain TBDM425 chromosome, complete genome	89	4411143	LAM
GCF_002447495.1	ASM244749v1	NZ_CP023604.1	Mycobacterium tuberculosis strain LE76 chromosome, complete genome	90	4411211	LAM
GCF_002447835.1	ASM244783v1	NZ_CP023621.1	Mycobacterium tuberculosis strain LN2900 chromosome, complete genome	91	4411327	LAM
GCF_002446915.1	ASM244691v1	NZ_CP023575.1	Mycobacterium tuberculosis strain CSV5769 chromosome, complete genome	92	4411312	Euro-American (4.9)
GCF_002447375.1	ASM244737v1	NZ_CP023598.1	Mycobacterium tuberculosis strain SLM100 chromosome, complete genome	93	4411394	Haarlem
GCF_002447675.1	ASM244767v1	NZ_CP023613.1	Mycobacterium tuberculosis strain LN3584 chromosome, complete genome	94	4411326	LAM
GCF_002447815.1	ASM244781v1	NZ_CP023620.1	Mycobacterium tuberculosis strain LN1856 chromosome, complete genome	95	4411299	LAM
GCF_002447875.1	ASM244787v1	NZ_CP023623.1	Mycobacterium tuberculosis strain MDRMA203 chromosome, complete genome	96	4411290	Haarlem
GCF_002447915.1	ASM244791v1	NZ_CP023625.1	Mycobacterium tuberculosis strain MDRMA863 chromosome, complete genome	97	4411432	Haarlem
GCF_000008585.1	ASM858v1	NC_002755.2	Mycobacterium tuberculosis CDC1551, complete genome	98	4403837	Euro-American (4.1.1.3)
GCF_000422125.1	ASM42212v1	NC_021740.1	Mycobacterium tuberculosis EAI5, complete genome	99	4391174	EAI
GCF_001855255.1	ASM185525v1	NZ_CP013475.1	Mycobacterium tuberculosis strain 1458, complete genome	100	4402033	Beijing
GCF_001895845.1	ASM189584v1	NZ_CP018300.1	Mycobacterium tuberculosis strain I0002353-6, complete genome	101	4385578	LAM
GCF_002447275.1	ASM244727v1	NZ_CP023593.1	Mycobacterium tuberculosis strain SLM040 chromosome, complete genome	102	4411408	Haarlem
GCF_002447535.1	ASM244753v1	NZ_CP023606.1	Mycobacterium tuberculosis strain LE103 chromosome, complete genome	103	4411243	LAM
GCF_001275565.2	ASM127556v2	NZ_CP012506.2	Mycobacterium tuberculosis strain SCAID 187.0 chromosome, complete genome	104	4411829	Beijing

GCF_002446955.1	ASM244695v1	NZ_CP023577.1	Mycobacterium tuberculosis strain CSV11678 chromosome, complete genome	105	4411382	Haarlem
GCF_002447055.1	ASM244705v1	NZ_CP023582.1	Mycobacterium tuberculosis strain LN3756 chromosome, complete genome	106	4411315	Euro-American (4.9)
GCF_002447075.1	ASM244707v1	NZ_CP023583.1	Mycobacterium tuberculosis strain MDRDM260 chromosome, complete genome	107	4411280	Euro-American (4.9)
GCF_002447135.1	ASM244713v1	NZ_CP023586.1	Mycobacterium tuberculosis strain MDRMA2491 chromosome, complete genome	108	4411121	Beijing
GCF_002447315.1	ASM244731v1	NZ_CP023595.1	Mycobacterium tuberculosis strain SLM060 chromosome, complete genome	109	4411134	Beijing
GCF_002447335.1	ASM244733v1	NZ_CP023596.1	Mycobacterium tuberculosis strain SLM063 chromosome, complete genome	110	4411337	Haarlem
GCF_002447355.1	ASM244735v1	NZ_CP023597.1	Mycobacterium tuberculosis strain SLM088 chromosome, complete genome	111	4411385	Haarlem
GCF_002447395.1	ASM244739v1	NZ_CP023599.1	Mycobacterium tuberculosis strain CSV383 chromosome, complete genome	112	4411115	Beijing
GCF_002447435.1	ASM244743v1	NZ_CP023601.1	Mycobacterium tuberculosis strain CSV9577 chromosome, complete genome	113	4411230	LAM
GCF_002447555.1	ASM244755v1	NZ_CP023607.1	Mycobacterium tuberculosis strain LE371 chromosome, complete genome	114	4411137	LAM
GCF_002447595.1	ASM244759v1	NZ_CP023609.1	Mycobacterium tuberculosis strain LN55 chromosome, complete genome	115	4411186	Beijing
GCF_002447935.1	ASM244793v1	NZ_CP023626.1	Mycobacterium tuberculosis strain MDRMA1565 chromosome, complete genome	116	4411159	Beijing
GCF_002448195.1	ASM244819v1	NZ_CP023639.1	Mycobacterium tuberculosis strain TBV4768 chromosome, complete genome	117	4411173	Beijing
GCF_000193185.2	ASM19318v2	NZ_CP012090.1	Mycobacterium tuberculosis W-148, complete genome	118	4418548	Beijing
GCF_000270365.1	ASM27036v1	NC_017522.1	Mycobacterium tuberculosis CCDC5180, complete genome	119	4405981	Beijing
GCF_000828995.1	ASM82899v1	NZ_AP014573.1	Mycobacterium tuberculosis str. Kurono DNA, complete genome	120	4415078	Euro-American (4.9)
GCF_000224435.1	ASM22443v1	NC_017524.1	Mycobacterium tuberculosis CTRI-2, complete genome	121	4398525	LAM
GCF_000756545.1	ASM75654v1	NZ_CP009427.1	Mycobacterium tuberculosis strain 96121, complete genome	122	4410945	EAI Manila
GCF_002116815.1	ASM211681v1	NZ_CP017596.1	Mycobacterium tuberculosis strain Beijing/391 chromosome, complete genome	123	4406925	Beijing
GCF_000364825.1	ASM36482v1	NC_021054.1	Mycobacterium tuberculosis str. Beijing/NITR203, complete genome	124	4411128	Beijing
GCF_000389945.1	ASM38994v1	NC_021194.1	Mycobacterium tuberculosis EAI5/NITR206, complete genome	125	4390306	EAI

GCF_001895865.1	ASM189586v1	NZ_CP018304.1	Mycobacterium tuberculosis strain M0002959-6, complete genome	126	4386447	LAM	
GCF_000572175.1	ASM57217v1	NZ_CP002883.1	Mycobacterium tuberculosis BT1, complete genome	127	4399405	Beijing	
GCF_001702435.1	ASM170243v1	NZ_CP016794.1	Mycobacterium tuberculosis strain SCAID 320.0 chromosome, complete genome	128	4406628	Beijing	
GCF_001895765.1	ASM189576v1	NZ_CP018303.1	Mycobacterium tuberculosis strain I0004241-1, complete genome	129	4386132	LAM	
GCF_002447095.1	ASM244709v1	NZ_CP023584.1	Mycobacterium tuberculosis strain MDRDM627 chromosome, complete genome	130	4411215	LAM	
GCF_000023625.1	ASM2362v1	NC_012943.1	Mycobacterium tuberculosis KZN 1435, complete genome	131	4398250	LAM	
GCF_000154585.2	ASM15458v2	NC_016768.1	Mycobacterium tuberculosis KZN 4207, complete genome	132	4394985	LAM	
GCF_000154605.2	ASM15460v2	NC_018078.1	Mycobacterium tuberculosis KZN 605, complete genome	133	4399120	LAM	
GCF_000698475.1	ASM69847v1	NZ_CP007803.1	Mycobacterium tuberculosis K, complete genome	134	4385518	Beijing	
GCF_000756525.1	ASM75652v1	NZ_CP009426.1	Mycobacterium tuberculosis strain 96075, complete genome	135	4379376	Beijing	
GCF_001544955.1	ASM154495v1	NZ_CP010337.1	Mycobacterium tuberculosis strain 22115, complete genome	136	4401829	New-1	
GCF_001895785.1	ASM189578v1	NZ_CP018305.1	Mycobacterium tuberculosis strain M0018684-2, complete genome	137	4359825	LAM	
GCF_001895805.1	ASM189580v1	NZ_CP018302.1	Mycobacterium tuberculosis strain I0004000-1, complete genome	138	4365724	LAM	
GCF_002447235.1	ASM244723v1	NZ_CP023591.1	Mycobacterium tuberculosis strain TBV5365 chromosome, complete genome	139	4411398	Euro-American (4.9)	
GCF_002447615.1	ASM244761v1	NZ_CP023610.1	Mycobacterium tuberculosis strain LN317 chromosome, complete genome	140	4411340	Euro-American (4.9)	
GCF_000350205.1	ASM35020v1	NC_020559.1	Mycobacterium tuberculosis str. Erdman = ATCC 35801 DNA, complete genome	141	4392353	Haarlem	
GCF_000831245.1	ASM83124v1	NZ_CP009480.1	Mycobacterium tuberculosis H37Rv, complete genome	142	4396119	Euro-American (4.9)	
GCF_001750865.1	ASM175086v1	NZ_CP011510.1	Mycobacterium tuberculosis strain Beijing, complete genome	143	4378588	Beijing	
GCF_001870145.1	ASM187014v1	NZ_CP017920.1	Mycobacterium tuberculosis strain TB282 chromosome, complete genome	144	4425860	Beijing	yes - TB282
GCF_001895825.1	ASM189582v1	NZ_CP018301.1	Mycobacterium tuberculosis strain I0002801-4, complete genome	145	4376067	Euro-American (4.1.1.3)	
GCF_002116855.1	ASM211685v1	NZ_CP017598.1	Mycobacterium tuberculosis strain Beijing-like/1104 chromosome, complete genome	146	4380156	Beijing	
