# Supplementary material for: Computational pan-genome mapping and pairwise SNP-distance improve detection of Mycobacterium tuberculosis transmission clusters
Source: PLoS Comput Biol. 2019 Dec 9;15(12):e1007527. doi: 10.1371/journal.pcbi.1007527 (PMC6922483; doi:10.1371/journal.pcbi.1007527)
Supplement: S8 Table — provides filtered genes with coordinates for the M. tuberculosis H37Rv genome and mapped to the computational pan-genome. (PDF) [file pcbi.1007527.s010.pdf]

GeneID	Symbol	Aliases	Description	H37Rv		pan-genome		
				Start	End	SegmentID	Start	End
887049	Rv0031	Rv0031	Possible remnant of a transposase	33582	33794	1	36422	36634
886938	PPE1	Rv0096	PPE family protein PPE1	105324	106715	2	108638	110029
886912	PE_PGRS1	Rv0109	PE-PGRS family protein PE_PGRS1	131382	132872	3	134701	136207
886883	PE_PGRS2	Rv0124	PE-PGRS family protein PE_PGRS2	149533	150996	4	152871	154623
886857	PE1	Rv0151c	PE family protein PE1	177543	179309	5	181279	183045
886838	PE2	Rv0152c	PE family protein PE2	179319	180896	6	183055	184632
886826	PE3	Rv0159c	PE family protein PE3	187433	188839	7	191170	192576
886825	PE4	Rv0160c	PE family protein PE4	188931	190439	8	192668	194176
886684	PPE2	Rv0256c	PPE family protein PPE2	307877	309547	9	344783	346455
886623	Rv0278c	Rv0278c	PE-PGRS family protein PE_PGRS3	333437	336310	10	370354	380011
886621	PE_PGRS4	Rv0279c	PE-PGRS family protein PE_PGRS4	336560	339073	11	380312	383320
886619	PPE3	Rv0280	PPE family protein PPE3	339364	340974	12	383615	385266
886608	PE5	Rv0285	PE family protein PE5	349624	349932	13	393920	394228
886607	PPE4	Rv0286	PPE family protein PPE4	349935	351476	14	394231	395773
885981	PE_PGRS5	Rv0297	PE-PGRS family protein PE_PGRS5	361334	363109	15	405632	407456
886592	PPE5	Rv0304c	PPE family protein PPE5	366150	372764	16	410498	417114
885978	PPE6	Rv0305c	PPE family protein PPE6	372820	375711	17	417170	420092
886527	PE6	Rv0335c	PE family protein PE6	399535	400050	18	443920	444435
886498	PPE7	Rv0354c	PPE family protein PPE7	424269	424694	19	468763	469189
886491	PPE8	Rv0355c	PPE family protein PPE8	424777	434679	20	469272	479222
886436	Rv0387c	Rv0387c	pseudo	466672	468001	21	512589	513920
886439	PPE9	Rv0388c	PPE family protein PPE9	467459	468001	21	512589	513920
886340	PPE10	Rv0442c	PPE family protein PPE10	530751	532214	22	578361	579839
886317	PPE11	Rv0453	PPE family protein PPE11	543174	544730	23	590803	592359
887391	PE_PGRS6	Rv0532	PE-PGRS family protein PE_PGRS6	622793	624577	24	672718	674895
887725	PE_PGRS7	Rv0578c	PE-PGRS family protein PE_PGRS7	671996	675916	25	722329	727115
888644	Rv0741	Rv0741	Probable transposase (fragment)	832534	832848	26	884616	884930
888645	Rv0742	Rv0742	hypothetical protein	832981	833508	27	885063	885591
888664	PE_PGRS9	Rv0746	PE-PGRS family protein PE_PGRS9	835701	838052	28	887784	890552
888662	PE_PGRS10	Rv0747	PE-PGRS family protein PE_PGRS10	838451	840856	29	890951	904025
888695	PE_PGRS11	Rv0754	PE-PGRS family protein PE_PGRS11	846159	847913	30	909340	911094

888708	PPE12	Rv0755c	PPE family protein PPE12	848103	850040		31	911284	913252
3205072	Rv0755A	Rv0755A	Putative transposase (fragment)	850342	850527		32	915450	915635
885454	Rv0795	Rv0795	insertion sequence element IS6110 transposase (fragment)	889072	889398		33	959541	960802
885099	Rv0796	Rv0796	insertion sequence element IS986/IS6110 transposase	889347	890333		33	959541	960802
885476	Rv0797	Rv0797	insertion sequence element IS1547 transposase	890388	891482		34	960884	961980
885236	PE_PGRS12	Rv0832	PE-PGRS family protein PE_PGRS12	924951	925364		35	995916	999047
885391	PE_PGRS13	Rv0833	PE-PGRS family protein PE_PGRS13	925361	927610		35	995916	999047
885369	PE_PGRS14	Rv0834c	PE-PGRS family protein PE_PGRS14	927837	930485		36	999274	1002402
885054	Rv0850	Rv0850	Putative transposase (fragment)	947312	947644		37	1024532	1024864
885742	PE_PGRS15	Rv0872c	PE-PGRS family protein PE_PGRS15	968424	970244		38	1045956	1047797
885617	PPE13	Rv0878c	PPE family protein PPE13	976872	978203		39	1054425	1055770
885069	PPE14	Rv0915c, MTE	PPE family protein PPE14	1020058	1021329		40	1097640	1098911
885167	PE7	Rv0916c, MTE	PE family protein PE7	1021344	1021643		41	1098926	1099225
885549	Rv0920c	Rv0920c	transposase	1025497	1026816		42	1103079	1104399
885564	Rv0922	Rv0922	transposase	1027685	1029337		43	1105268	1106921
885264	PE_PGRS16	Rv0977	PE-PGRS family protein PE_PGRS16	1090373	1093144		44	1172555	1175357
885077	PE_PGRS17	Rv0978c	PE-PGRS family protein PE_PGRS17	1093361	1094356		45	1175574	1176875
885327	PE_PGRS18	Rv0980c	PE-PGRS family protein PE_PGRS18	1095078	1096451		46	1177597	1179456
886010	Rv1034c	Rv1034c	Probable transposase (fragment)	1158918	1159307		47	1241948	1242337
888206	Rv1035c	Rv1035c	Probable transposase (fragment)	1159375	1160061		48	1242405	1243092
888227	Rv1036c	Rv1036c	Probable IS1560 transposase (fragment)	1160095	1160433		49	1243126	1243464
888477	PPE15	Rv1039c	PPE family protein PPE15	1161297	1162472		50	1244328	1245505
888533	PE8	Rv1040c	PE family protein PE8	1162549	1163376		51	1245582	1247767
888546	Rv1041c	Rv1041c	IS2-like transposase	1164572	1165435		52	1248963	1249890
888607	Rv1042c	Rv1042c	IS2-like transposase	1165092	1165499		52	1248963	1249890
886060	Rv1047	Rv1047	transposase	1169423	1170670		53	1254003	1255250
887139	Rv1054	Rv1054	Probable integrase (fragment)	1176928	1177242		54	1262871	1263185
887122	PE_PGRS19	Rv1067c	PE-PGRS family protein PE_PGRS19	1188421	1190424		55	1274366	1277792
887123	PE_PGRS20	Rv1068c	PE-PGRS family protein PE_PGRS20	1190757	1192148		56	1278125	1280512

887094	PE_PGRS21	Rv1087	PE-PGRS family protein PE_PGRS21	1211560	1213863	57	1299926	1302791
887096	PE9	Rv1088	PE family protein PE9	1214513	1214947	58	1303442	1304060
887090	PE10	Rv1089	PE family protein PE10	1214769	1215131	58	1303442	1304060
885258	PE_PGRS22	Rv1091	PE-PGRS family protein PE_PGRS22	1216469	1219030	59	1305422	1308861
885131	PPE16	Rv1135c	PPE family protein PPE16	1262272	1264128	60	1352154	1355377
885164	Rv1149	Rv1149	transposase	1277893	1278300	61	1369147	1369554
885990	PPE17	Rv1168c	PPE family protein PPE17	1298764	1299804	62	1390024	1391064
885930	lipX	Rv1169c, PE1	lipase LipX	1299822	1300124	63	1391082	1391384
885988	PE12	Rv1172c	PE family protein PE12	1301755	1302681	64	1393017	1393943
886044	PE13	Rv1195	PE family protein PE13	1339003	1339302	65	1430339	1430638
886073	PPE18	Rv1196, mtb3	PPE family protein PPE18	1339349	1340524	66	1430685	1431866
886092	Rv1199c	Rv1199c	insertion sequence element IS1081 transposase	1341358	1342605	67	1432749	1433996
888362	PE14	Rv1214c	PE family protein PE14	1357293	1357625	68	1448689	1449021
887109	PE_PGRS23	Rv1243c	PE-PGRS family protein PE_PGRS23	1384989	1386677	69	1476396	1478131
886922	Rv1313c	Rv1313c	insertion sequence element IS1557 transposase	1468171	1469505	70	1564143	1565477
886899	PE_PGRS24	Rv1325c	PE-PGRS family protein PE_PGRS24	1488154	1489965	71	1588017	1590841
886819	PPE19	Rv1361c, mtb	PPE family protein PPE19	1532443	1533633	72	1637397	1638592
886789	Rv1369c	Rv1369c	insertion sequence element IS986/IS6110 transposase	1541994	1542980	73	1646962	1648223
886791	Rv1370c	Rv1370c	insertion sequence element IS6110 transposase(fragment)	1542929	1543255	73	1646962	1648223
886757	PE15	Rv1386	PE family protein PE15	1561464	1561772	74	1669168	1671092
886784	PPE20	Rv1387	PPE family protein PPE20	1561769	1563388	74	1669168	1671092
886745	PE_PGRS25	Rv1396c	PE-PGRS family protein PE_PGRS25	1572127	1573857	75	1679831	1681571
886652	PE16	Rv1430	PE family protein PE16	1606386	1607972	76	1714109	1715695
886626	PE_PGRS26	Rv1441c	PE-PGRS family protein PE_PGRS26	1618209	1619684	77	1725986	1727528
886605	PE_PGRS27	Rv1450c	PE-PGRS family protein PE_PGRS27	1630638	1634627	78	1738490	1744196
886595	PE_PGRS28	Rv1452c	PE-PGRS family protein PE_PGRS28	1636004	1638229	79	1745573	1748163
886556	PE_PGRS29	Rv1468c	PE-PGRS family protein PE_PGRS29	1655609	1656721	80	1765596	1769384
886384	PPE21	Rv1548c	PPE family protein PPE21	1751297	1753333	81	1895264	1897300
886337	Rv1573	Rv1573	phage protein	1779314	1779724	82	1926323	1926789

886331	Rv1574	Rv1574	phage protein	1779930	1780241	83	1926995	1929130
886335	Rv1575	Rv1575	phage protein	1780199	1780699	83	1926995	1929130
886327	Rv1576c	Rv1576c	phage capsid protein	1780643	1782064	83	1926995	1929130
886329	Rv1577c	Rv1577c	phage prohead protease	1782072	1782584	84	1929138	1929650
886322	Rv1578c	Rv1578c	phage protein	1782758	1783228	85	1929824	1930294
886369	Rv1579c	Rv1579c	phage protein	1783309	1783623	86	1930375	1930958
886313	Rv1580c	Rv1580c	phage protein	1783620	1783892	86	1930375	1930958
886318	Rv1581c	Rv1581c	phage protein	1783906	1784301	87	1930972	1931367
886311	Rv1582c	Rv1582c	phage protein	1784497	1785912	88	1931563	1933594
886315	Rv1583c	Rv1583c	phage protein	1785912	1786310	88	1931563	1933594
886307	Rv1584c	Rv1584c	phage protein	1786307	1786528	88	1931563	1933594
886309	Rv1585c	Rv1585c	phage protein	1786584	1787099	89	1933650	1935571
886305	Rv1586c	Rv1586c	phage integrase	1787096	1788505	89	1933650	1935571
885486	PE17	Rv1646	PE family protein PE17	1855764	1856696	90	2116522	2117454
885174	PE_PGRS30	Rv1651c	PE-PGRS family protein PE_PGRS30	1862347	1865382	91	2123105	2126165
885068	PPE22	Rv1705c	PPE family protein PPE22	1931497	1932654	92	2197800	2198957
885070	PPE23	Rv1706c	PPE family protein PPE23	1932694	1933878	93	2198997	2200181
885544	PPE24	Rv1753c	PPE family protein PPE24	1981614	1984775	94	2251185	2256637
885541	Rv1756c	Rv1756c	Putative transposase	1987745	1988731	95	2270615	2271876
885558	Rv1757c	Rv1757c	Putative transposase for insertion sequence element IS6110 (fragment)	1988680	1989006	95	2270615	2271876
885372	Rv1763	Rv1763	Putative transposase for insertion sequence element IS6110 (fragment)	1996152	1996478	96	2287069	2290574
885238	Rv1764	Rv1764	Putative transposase	1996427	1997413	96	2287069	2290574
3205098	Rv1765A	Rv1765A	Putative transposase (fragment)	1999142	1999357	97	2293963	2294178
885429	PE_PGRS31	Rv1768	PE-PGRS family protein PE_PGRS31	2000614	2002470	98	2295435	2297319
885827	PPE25	Rv1787	PPE family protein PPE25	2025301	2026398	99	2321511	2322610
885895	PE18	Rv1788	PE family protein PE18	2026477	2026776	100	2322689	2324346
885333	PPE26	Rv1789	PPE family protein PPE26	2026790	2027971	101	2324360	2325541
885859	PPE27	Rv1790	PPE family protein PPE27	2028425	2029477	102	2325995	2327049
885445	PE19	Rv1791	PE family protein PE19	2029904	2030203	103	2327476	2327775
885465	PPE28	Rv1800	PPE family protein PPE28	2039453	2041420	104	2339774	2343892

885491	PPE29	Rv1801	PPE family protein PPE29	2042001	2043272	105	2345831	2347104
885542	PPE30	Rv1802	PPE family protein PPE30	2043384	2044775	106	2347216	2349965
885730	PE_PGRS32	Rv1803c	PE-PGRS family protein PE_PGRS32	2044923	2046842	107	2350113	2352043
885537	PE20	Rv1806	PE family protein PE20	2048072	2048371	108	2354631	2354930
885072	PPE31	Rv1807	PPE family protein PPE31	2048398	2049597	109	2354957	2356157
885590	PPE32	Rv1808	PPE family protein PPE32	2049921	2051150	110	2356483	2357712
885555	PPE33	Rv1809	PPE family protein PPE33	2051282	2052688	111	2357844	2359250
885551	PE_PGRS33	Rv1818c	PE-PGRS family protein PE_PGRS33	2061178	2062674	112	2367757	2369313
885753	PE_PGRS34	Rv1840c	PE-PGRS family protein PE_PGRS34	2087971	2089518	113	2396374	2397927
885362	PPE34	Rv1917c	PPE family protein PPE34	2162932	2167311	114	2471535	2484365
885506	PPE35	Rv1918c	PPE family protein PPE35	2167649	2170612	115	2484733	2487701
885921	PE_PGRS35	Rv1983	PE-PGRS family protein PE_PGRS35	2226244	2227920	116	2555194	2556880
887546	Rv2013	Rv2013	Transposase	2260665	2261144	117	2589659	2590682
887547	Rv2014	Rv2014	Transposase	2261098	2261688	117	2589659	2590682
888395	Rv2105	Rv2105	Putative transposase for insertion sequence element IS6110 (fragment)	2365465	2365791	118	2714913	2716174
888398	Rv2106	Rv2106	Probable transposase	2365740	2366726	118	2714913	2716174
887811	PE22	Rv2107	PE family protein PE22	2367359	2367655	119	2716819	2717115
887814	PPE36	Rv2108	PPE family protein PPE36	2367711	2368442	120	2717171	2723171
888710	PPE37	Rv2123, irg2	PPE family protein PPE37	2381071	2382492	121	2736032	2737453
887791	PE_PGRS37	Rv2126c	PE-PGRS family protein PE_PGRS37	2387202	2387972	122	2742163	2742934
887300	PE_PGRS38	Rv2162c	PE-PGRS family protein PE_PGRS38	2423240	2424838	123	2778334	2779963
888197	Rv2167c	Rv2167c	insertion sequence element IS986/IS6110 transposase	2430159	2431145	124	2785285	2786546
888459	Rv2168c	Rv2168c	Putative transposase for insertion sequence element IS6110 (fragment)	2431094	2431420	124	2785285	2786546
888326	Rv2177c	Rv2177c	transposase	2439282	2439947	125	2794425	2795090
888602	Rv2278	Rv2278	insertion sequence element IS6110 transposase(fragment)	2550065	2550391	126	2909783	2911044
887746	Rv2279	Rv2279	insertion sequence element IS986/IS6110 transposase	2550340	2551326	126	2909783	2911044
888111	PE23	Rv2328	PE family protein PE23	2600731	2601879	127	2967174	2968322
888961	PE_PGRS39	Rv2340c	PE-PGRS family protein PE_PGRS39	2617667	2618908	128	2990997	2992238

888959	PPE38	Rv2352c	PPE family protein PPE38	2632923	2634098	129	3018365	3019544
886003	PPE39	Rv2353c	PPE family protein PPE39	2634528	2635592	130	3019976	3024138
888963	Rv2354	Rv2354	insertion sequence element IS6110 transposase(fragment)	2635628	2635954	131	3024186	3025447
888957	Rv2355	Rv2355	insertion sequence element IS986/IS6110 transposase	2635903	2636889	131	3024186	3025447
888950	PPE40	Rv2356c	PPE family protein PPE40	2637688	2639535	132	3026276	3030216
885141	PE_PGRS40	Rv2371	PE-PGRS family protein PE_PGRS40	2651753	2651938	133	3042435	3042620
885517	PE_PGRS41	Rv2396, aprC	acid and phagosome regulated protein AprC	2692799	2693884	134	3084909	3086014
885511	PE24	Rv2408	PE family protein PE24	2705762	2706736	135	3097917	3098891
885699	Rv2424c	Rv2424c	transposase	2720776	2721777	136	3112937	3113938
885945	PPE41	Rv2430c	PPE family protein PPE41	2727336	2727920	137	3119497	3120081
885703	PE25	Rv2431c	PE family protein PE25	2727967	2728266	138	3120128	3120427
887201	Rv2479c	Rv2479c	insertion sequence element IS986/IS6110 transposase	2784657	2785643	139	3176839	3178100
887328	Rv2480c	Rv2480c	insertion sequence element IS6110 transposase(fragment)	2785592	2785918	139	3176839	3178100
887909	PE_PGRS42	Rv2487c	PE-PGRS family protein PE_PGRS42	2795301	2797385	140	3187495	3189585
887941	PE_PGRS43	Rv2490c	PE-PGRS family protein PE_PGRS43	2801254	2806236	141	3193454	3199797
888515	Rv2512c	Rv2512c	insertion sequence element IS1081 transposase	2828556	2829803	142	3236142	3237389
888172	PE26	Rv2519	PE family protein PE26	2835785	2837263	143	3243376	3244854
887992	PE_PGRS44	Rv2591	PE-PGRS family protein PE_PGRS44	2921551	2923182	144	3329839	3331480
888204	PPE42	Rv2608	PPE family protein PPE42	2935046	2936788	145	3343347	3345089
888215	PE_PGRS45	Rv2615c	PE-PGRS family protein PE_PGRS45	2943600	2944985	146	3351902	3362479
888573	PE_PGRS46	Rv2634c	PE-PGRS family protein PE_PGRS46	2960105	2962441	147	3377604	3379950
887706	Rv2646	Rv2646	integrase	2970551	2971549	148	3388062	3389060
887828	Rv2648	Rv2648	insertion sequence element IS6110 transposase(fragment)	2972160	2972486	149	3389758	3391020
888553	Rv2649	Rv2649	insertion sequence element IS986/IS6110 transposase	2972435	2973421	149	3389758	3391020
887478	Rv2650c	Rv2650c	prophage protein	2973795	2975234	150	3391397	3392839

887837	Rv2651c	Rv2651c	prophage protease	2975242	2975775		151	3392847	3393380
888577	Rv2652c	Rv2652c	prophage protein	2975928	2976554		152	3393541	3394167
887367	Rv2653c	Rv2653c	toxin	2976586	2976909		153	3394200	3394523
888154	Rv2654c	Rv2654c	antitoxin	2976989	2977234		154	3394603	3396272
887388	Rv2655c	Rv2655c	prophage protein	2977231	2978658		154	3394603	3396272
888179	Rv2656c	Rv2656c	prophage protein	2978660	2979052		155	3396274	3396923
887399	Rv2657c	Rv2657c	prophage protein	2979049	2979309		155	3396274	3396923
885098	Rv2659c	Rv2659c	prophage integrase	2979691	2980818		156	3397305	3398432
888904	Rv2666	Rv2666	Probable transposase for insertion sequence element IS1081 (fragment)	2983071	2983874		157	3402044	3402847
888339	PE_PGRS47	Rv2741	PE-PGRS family protein PE_PGRS47	3053914	3055491		158	3486888	3488471
887765	PPE43	Rv2768c	PPE family protein PPE43	3076894	3078078		159	3511236	3512420
888461	PE27	Rv2769c	PE family protein PE27	3078158	3078985		160	3512500	3513328
888456	PPE44	Rv2770c	PPE family protein PPE44	3079309	3080457		161	3513652	3514800
888281	Rv2791c	Rv2791c	transposase	3100202	3101581		162	3537266	3538645
887784	Rv2810c	Rv2810c	Probable transposase	3115741	3116142		163	3555527	3555928
888942	Rv2812	Rv2812	transposase	3116818	3118227		164	3556604	3558013
887839	Rv2814c	Rv2814c	insertion sequence element IS986/IS6110 transposase	3120566	3121552		165	3564995	3566256
888511	Rv2815c	Rv2815c	insertion sequence element IS6110 transposase(fragment)	3121501	3121827		165	3564995	3566256
888171	PE_PGRS48	Rv2853	PE-PGRS family protein PE_PGRS48	3162268	3164115		166	3612510	3614399
887173	Rv2885c	Rv2885c	transposase	3194166	3195548		167	3644669	3646055
887824	PPE45	Rv2892c	PPE family protein PPE45	3200794	3202020		168	3651303	3652530
887834	Rv2943	Rv2943	insertion sequence element IS1533 transposase	3288464	3289705		169	3739862	3741904
3205061	Rv2943A	Rv2943A	transposase	3289705	3290235		169	3739862	3741904
887636	Rv2944	Rv2944	insertion sequence element IS1533 transposase	3289790	3290506		169	3739862	3741904
887316	Rv2961	Rv2961	transposase	3313283	3313672		170	3766227	3766616
887390	Rv2978c	Rv2978c	transposase	3333785	3335164		171	3786735	3788114
888940	PPE46	Rv3018c	PPE family protein PPE46	3376939	3378243		172	3830927	3832245
3205087	PE27A	Rv3018A	PE family protein PE27A	3378329	3378415		173	3832331	3832423

888924	PPE47	Rv3021c	pseudo	3379376	3380452	174	3843448	3850569
888512	PPE48	Rv3022c	pseudo	3380440	3380682	174	3843448	3850569
3205088	PE29	Rv3022A	PE family protein PE29	3380679	3380993	174	3843448	3850569
888525	Rv3023c	Rv3023c	transposase	3381375	3382622	175	3851607	3853379
888790	Rv3115	Rv3115	transposase	3481451	3482698	176	3954127	3955374
888892	PPE49	Rv3125c	PPE family protein PPE49	3490476	3491651	177	3964511	3968404
888153	PPE50	Rv3135	PPE family protein PPE50	3501334	3501732	178	3980869	3982608
888835	PPE51	Rv3136	PPE family protein PPE51	3501794	3502936	179	3982670	3983812
887930	PPE52	Rv3144c	PPE family protein PPE52	3510088	3511317	180	3990965	3992194
888794	PPE53	Rv3159c	PPE family protein PPE53	3527391	3529163	181	4008272	4012224
888796	Rv3184	Rv3184	insertion sequence element IS6110 transposase(fragment)	3551281	3551607	182	4041274	4042535
887441	Rv3185	Rv3185	insertion sequence element IS986/IS6110 transposase	3551556	3552542	182	4041274	4042535
888024	Rv3186	Rv3186	insertion sequence element IS6110 transposase(fragment)	3552764	3553090	183	4042764	4044025
887604	Rv3187	Rv3187	insertion sequence element IS986/IS6110 transposase	3553039	3554025	183	4042764	4044025
887628	Rv3191c	Rv3191c	transposase	3557311	3558345	184	4048748	4049782
887314	Rv3325	Rv3325	insertion sequence element IS6110 transposase(fragment)	3710433	3710759	185	4213364	4214625
887563	Rv3326	Rv3326	insertion sequence element IS986/IS6110 transposase	3710708	3711694	185	4213364	4214625
887965	Rv3327	Rv3327	transposase fusion protein	3711749	3713461	186	4214953	4222316
888033	PPE54	Rv3343c	PPE family protein PPE54	3729364	3736935	187	4239582	4253069
888115	PE_PGRS49	Rv3344c	PE-PGRS family protein PE_PGRS49	3736984	3738000	188	4253118	4254308
888114	PE_PGRS50	Rv3345c	PE-PGRS family protein PE_PGRS50	3738158	3742774	189	4254699	4263661
888120	PPE55	Rv3347c	PPE family protein PPE55	3743711	3753184	190	4264600	4275439
888110	Rv3348	Rv3348	transposase	3753765	3754256	191	4276020	4276511
888126	Rv3349c	Rv3349c	transposase	3754293	3755237	192	4276548	4277492
888113	PPE56	Rv3350c	PPE family protein PPE56	3755952	3767102	193	4278207	4289375
887404	PE_PGRS51	Rv3367	PE-PGRS family protein PE_PGRS51	3778568	3780334	194	4300842	4302628

887411	Rv3380c	Rv3380c	insertion sequence element IS986/IS6110 transposase	3795100	3796086	195	4317399	4318660
887646	Rv3381c	Rv3381c	insertion sequence element IS6110 transposase(fragment)	3796035	3796361	195	4317399	4318660
888044	Rv3386	Rv3386	transposase	3800092	3800796	196	4325289	4326660
887820	Rv3387	Rv3387	transposase	3800786	3801463	196	4325289	4326660
888151	PE_PGRS52	Rv3388	PE-PGRS family protein PE_PGRS52	3801653	3803848	197	4326850	4329423
887635	PPE57	Rv3425	PPE family protein PPE57	3842239	3842769	198	4368876	4369406
887622	PPE58	Rv3426	PPE family protein PPE58	3843036	3843734	199	4369673	4370383
887631	Rv3427c	Rv3427c	transposase	3843885	3844640	200	4370534	4371289
887621	Rv3428c	Rv3428c	transposase	3844738	3845970	201	4372756	4373990
887630	PPE59	Rv3429	PPE family protein PPE59	3847165	3847701	202	4397378	4399069
887615	Rv3430c	Rv3430c	transposase	3847642	3848805	202	4397378	4399069
888097	Rv3474	Rv3474	insertion sequence element IS6110 transposase(fragment)	3890830	3891156	203	4460421	4461683
888055	Rv3475	Rv3475	insertion sequence element IS986/IS6110 transposase	3891105	3892091	203	4460421	4461683
888474	PE31	Rv3477	PE family protein PE31	3894093	3894389	204	4472297	4472593
888047	PPE60	Rv3478, mtb3	PE family protein PPE60	3894426	3895607	205	4472630	4473816
888256	PE_PGRS53	Rv3507	PE-PGRS family protein PE_PGRS53	3926569	3930714	206	4504786	4611956
888270	PE_PGRS54	Rv3508	PE-PGRS family protein PE_PGRS54	3931005	3936710	207	4612247	4625244
888273	PE_PGRS55	Rv3511	PE-PGRS family protein PE_PGRS55	3939617	3941761	208	4628153	4631030
888306	PE_PGRS56	Rv3512	PE-PGRS family protein PE_PGRS56	3943812	3944963	209	4633925	4635114
888294	PE_PGRS57	Rv3514	PE-PGRS family protein PE_PGRS57	3945794	3950263	210	4635945	4641776
888370	PPE61	Rv3532	PPE family protein PPE61	3969343	3970563	211	4660862	4662082
888385	PPE62	Rv3533c	PPE family protein PPE62	3970705	3972453	212	4662224	4663978
888438	PPE63	Rv3539	PPE family protein PPE63	3978059	3979498	213	4669599	4671039
887822	PPE64	Rv3558	PPE family protein PPE64	3997980	3999638	214	4689528	4691186
887874	PE_PGRS58	Rv3590c	PE-PGRS family protein PE_PGRS58	4031404	4033158	215	4722955	4724996
885464	PE_PGRS59	Rv3595c	PE-PGRS family protein PE_PGRS59	4036731	4038050	216	4728569	4729925
885097	PPE65	Rv3621c	PPE family protein PPE65	4060648	4061889	217	4753121	4754363
885712	PE32	Rv3622c	PE family protein PE32	4061899	4062198	218	4754373	4754672
885274	Rv3636	Rv3636	pseudo	4075752	4076984	219	4768239	4770217

885496	Rv3637	Rv3637	Possible transposase	4076484	4076984	219	4768239	4770217
885803	Rv3638	Rv3638	transposase	4076984	4077730	219	4768239	4770217
885324	Rv3640c	Rv3640c	transposase	4078520	4079749	220	4772555	4773784
885832	PE33	Rv3650	PE family protein PE33	4091233	4091517	221	4785333	4785617
886260	PE_PGRS60	Rv3652	PE-PGRS family-related protein PE_PGRS60	4093632	4093946	222	4787733	4788755
886259	PE_PGRS61	Rv3653	PE-PGRS family-related protein PE_PGRS61	4093940	4094527	222	4787733	4788755
886262	PPE66	Rv3738c	PPE family protein PPE66	4189285	4190232	223	4885358	4886305
886257	PPE67	Rv3739c	PPE family protein PPE67	4190284	4190517	224	4886357	4886590
885764	PE34	Rv3746c	PE family protein PE34	4196171	4196506	225	4892244	4892579
885857	Rv3751	Rv3751	Probable integrase (fragment)	4198874	4199089	226	4894952	4895167
886268	Rv3798	Rv3798	insertion sequence element IS1557 transposase	4252993	4254327	227	4949119	4950454
886143	PE_PGRS62	Rv3812	PE-PGRS family protein PE_PGRS62	4276571	4278085	228	4972700	4974214
886151	Rv3827c	Rv3827c	transposase	4301563	4302789	229	4997694	4998920
886180	Rv3844	Rv3844	transposase	4318775	4319266	230	5014923	5015414
886191	PE35	Rv3872	PE family protein PE35	4350745	4351044	231	5047064	5047363
886201	PPE68	Rv3873	PPE family protein PPE68	4351075	4352181	232	5047394	5048501
886227	PPE69	Rv3892c	PPE family protein PPE69	4374484	4375683	233	5070915	5072114
886213	PE36	Rv3893c	PE family protein PE36	4375762	4375995	234	5072193	5072427
